# Identification and classification of honey’s authenticity by attenuated total reflectance Fourier-transform infrared spectroscopy and chemometric method

**DOI:** 10.14202/vetworld.2019.1304-1310

**Published:** 2019-08-23

**Authors:** Muhamad Sahlan, Seffiani Karwita, Misri Gozan, Heri Hermansyah, Masafumi Yohda, Young Je Yoo, Diah Kartika Pratami

**Affiliations:** 1 Department of Chemical Engineering, Faculty of Engineering, Universitas Indonesia, Depok, West Java 16424, Indonesia; 2 Research Center for Biomedical Engineering, Faculty of Engineering, Universitas Indonesia, Depok, West Java 16424, Indonesia; 3 Department of Biotechnology and Life Science, Faculty of Engineering, Tokyo University of Agriculture and Technology, Koganei, Tokyo 184-8588, Japan; 4 Department of Chemical and Biological Engineering, Seoul National University, Seoul, Korea; 5 Laboratory of Pharmacognosy and Phytochemistry, Faculty of Pharmacy, Pancasila University, Jakarta 12640, Indonesia

**Keywords:** *Apis* spp, attenuated total reflectance Fourier transform infrared, discriminant, spectrum, *Tetragonula* spp

## Abstract

**Background and Aim:**

The authentication of honey is important to protect industry and consumers from such adulterated honey. However, until now, there has been no guarantee of honey’s authenticity, especially in Indonesia. The classification of honey is based on the bee species (spp.) that produces it. The study used honey from sting bee *Apis* spp. and stingless bee *Tetragonula* spp. based on the fact that the content off honey produced between them has differences. Authenticating honey with currently available rapid detection methods, such as ^13^C nuclear magnetic resonance analysis, is costly. This study aimed to develop an inexpensive, fast, precise, and accurate classification method for authenticating honey.

**Materials and Methods:**

In this study, we use attenuated total reflectance Fourier-transform infrared (ATR-FTIR) spectroscopy with wavelengths ranging between 550 and 4000 cm^−1^ as an alternative analysis method, which is relatively less expensive. The spectra of authentic and fake honey samples were obtained using ATR-FTIR and plotted using chemometric discriminant analysis. The authentic honey samples were acquired from a local Indonesian breeder of honey bees, while the fake honey samples were made from a mixture of water, sugar, sodium bicarbonate, and authentic honey. Data were collected using Thermo Scientific’s OMNIC FTIR software and processed using Thermo Scientific’s TQ Analyst software.

**Results:**

Our method effectively classified the honey as authentic or fraudulent based on the FTIR spectra. To authenticate the honey, we formed two classes: Real honey and fake honey. The wavelengths that can best differentiate between these two classes correspond to four regions: 1600-1700 cm^−1^; 1175-1540 cm^−1^; 940-1175 cm^−1^; and 700-940 cm^−1^. Similarly, for classification purpose, we formed two classes: *Apis* spp. and *Tetragonula* spp. The wavelength region that can best classify the samples as belonging to the *Apis* spp. or *Tetragonula* spp. class is explicitly within the range of 1600-1700 cm^−1^.

**Conclusion:**

This study successfully demonstrated a method to rapidly and accurately classify and authenticate honey. ATR-FTIR is a useful tool to test the authenticity of honey.

## Introduction

Honey is a complex compound derived from a process called nectar synthesis, in which honey bees collect nectar from plants [1]. Honey has well-known therapeutic potential, including wound healing and infection prevention [2]. Honey is very famous for its potential therapeutic role in the treatment of disease by phytochemical, antimicrobial, anti-inflammatory, and antioxidant properties [3]. There are three types of honey bees in Indonesia: Giant honey bees (*Apis dorsata*), beekeeping honey bees (*Apis cerana* and *Apis mellifera*), and stingless bees *Melipona* [2]. The content of honey produced by *Apis* spp. and stingless bee has some differences. Honey produced by *Apis* spp. has a sugar content of 62-70% and a water content of 14.86-17.53% [4]. Honey produced by stingless bee has a sugar content of 44.08% and a water content of 30%-35%. Due to their small size, stingless bee has a low honey yield per colony; thus, the cost of honey derived from stingless bees is much higher than that of honey derived from other bee species [5]. Many people consume honey due to the health benefits it provides [6], and the consumption of honey as a natural supplement has been on the rise over the past few decades. Unfortunately, the supply of honey in nature cannot meet this demand. According to the Ministry of Environment and Forestry in Indonesia, the demand for honey in Indonesia in 2015 reached 7500 tons/year, whereas the supply only reached 5000 tons/year. Exploitation of this supply-demand problem has led to the manufacture of adulterated and fake honey through the addition of other food ingredients [7]. For example, honey has been deliberately diluted with high-fructose corn syrup – an inexpensive sweetener that has been linked to diabetes. Adulation of honey not only deceives health-conscious consumers but also destabilizes economic markets by introducing unfair competition.

A rapid, sensitive, inexpensive, and accurate method to detect the authenticity of honey is critically needed to address this problem. However, currently available rapid detection methods do not provide a level of accuracy sufficient to perform authenticity validation [8]. Moreover, many of these methods have low levels of precision [9]. The authenticity of the botanical origin of bee products is typically determined by organoleptic sensory analysis, physicochemical methods, and pollen analysis [7]. The authenticity of the geographical region of origin is determined by melissopalynological characterization methods [7]. However, these analyses require specialized expertise and are time-consuming [7]. In our experiment, the authenticity of honey was tested using gas chromatography (GC) to analyze the presence of ethanol and carbon dioxide (CO_2_) [10]. Real honey should contain oxygen (O_2_) but not CO_2_. Hydrogen peroxide, a compound naturally found in honey, is unstable and decomposes to O_2_ and water (H_2_O). One case of the fake honey found that they use sodium bicarbonate (NaHCO_3_) to produce bubble (like fresh natural honey), which produces CO_2_ [10].

To analyze and authenticate honey samples, we developed an innovative method based on attenuated total reflectance Fourier-transform infrared (ATR-FTIR) spectroscopy and chemometric discriminant analysis. The advantage of this approach is that it relies on the fact that every chemical compound has a unique and specific IR spectrum. Thus, the obtained spectra are representative of the characteristics of an organic sample as a whole [11]. FTIR spectroscopy can be used to non-destructively and rapidly obtain biochemical fingerprints of samples [12]. The crystal used in ATR cells is made from zinc selenide (ZnSe), and its low solubility in water and very high refractive index enable more precise measurements [13]. Moreover, the ATR-FTIR method has several advantages – it is relatively inexpensive, provides rapid quantification, does not damage the sample, eliminates the need for sample preparation, and requires only a small sample amount to perform the measurements [14].

This study aimed to develop an inexpensive, fast, precise, and accurate classification method for authenticating honey.

## Materials and Methods

### Ethical approval

This *in vitro* study did not need ethical approval from the University Ethics Committee.

### FTIR samples

For this study, we used a total of 85 samples consisting of 58 samples of real honey and 27 samples of fake (adulterated) honey. Real honey was collected from different geographical regions of Indonesia and represented various floral origins. Most of the samples were collected directly from primary honey producers.

The samples of real honey were produced by *A. cerana* (n=5), *A. mellifera* (n=19), *A. dorsata* (n=17), *Tetragonula* spp. (n=10) (stingless bee), and other stingless bee species (n=7). The 27 fake honey samples were made by mixing real honey with water, sucrose, and NaHCO_3_. Honey samples were grouped as either real or fake for identification purposes. For classification purposes, the honey samples were labeled as *Apis* spp. and *Tetragonula* spp.

### Instrumentation and samples analysis

Spectra from all samples were collected with Nicolet iS5 FTIR spectrometer (Thermo Fisher Scientific Inc., Waltham, MA, USA) equipped with an iD3 ATR accessory component. OMNIC software version 9 (Thermo Fisher Scientific Inc., Waltham, MA, USA) was used for spectral data acquisition. Samples were placed on a diamond/ZnSe crystal plate (Thermo Fisher Scientific Inc., Waltham, MA, USA) and scanned at room temperature from 550 to 4000 cm^−1^ for a 16 scan time with a resolution of 16 cm^−1^. Measurements for each sample were replicated 3 times, and the resulting identical spectra were analyzed. This process was done to evaluate the absorbance value accuracy, which could be affected by sample homogeneity. Propanol was used to clean the diamond between each sample measurement.

### Chemometrics

Discriminant analysis with TQ Analyst software (Thermo Fisher Scientific Inc., Waltham, MA, USA) was performed to classify the samples based on spectral differences. In this work, discriminant analysis was performed for the determination of spectral differentiation. In this work, discriminant analysis was conducted on ATR-FTIR spectra with the following wavelength ranges: 2800-3000 cm^−1^, 1600-1700 cm^−1^, 1175-1540 cm^−1^, 940-1175 cm^−1^, and 700-940 cm^−1^.

## Results

### ATR-FTIR analysis

**Figure-1:**
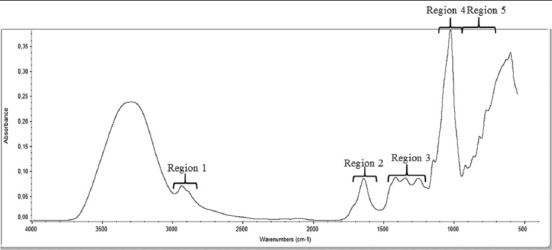
Representative of honey’s spectrum in the550-4000 cm^−1^ spectral region using attenuated total reflectance Fourier transform infrared.

**Figure-2:**
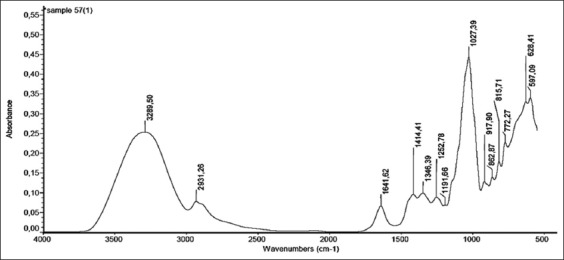
Attenuated total reflectance Fourier transform infrared spectrum for real honey in the 550-4000 cm^−1^ spectral region.

Figure-1 shows a real honey ATR-FTIR spectrum, which is divided into five specific regions. The band assignments and corresponding modes of vibration are shown in Table-1, based on Gok, 2014 [15]. The ATR-FTIR spectrum for real honey in the 550-4000 cm^−1^ spectral region is shown in Figure-2.

**Table 1:** General band assignment of ATR-FTIR spectrum of honey.

Region 1	2800-3000 cm^−1^	C-H stretching (carbohydrates)
		O-H stretching (carboxylic acids)
		NH_3_ stretching (free amino acids)
Region 2	1600-1700 cm^−1^	O-H stretching/bending (water)
		C=O stretching (mainly from carbohydrates)
		N-H bending of amide I (mainly proteins)
Region 3	1175-1540 cm^−1^	O-H stretching/bending
		C-O stretching (carbohydrates)
		C-H stretching (carbohydrates)
		C=O stretching of ketones
Region 4	940-1175 cm^−1^	C-O and C-C stretching (carbohydrates)
		Ring vibrations (mainly from carbohydrates)
Region 5	700-940 cm^−1^	Anomeric region of carbohydrates
		C-H bending (mainly from carbohydrates)
		Ring vibrations (mainly from carbohydrates)

ATR-FTIR=Attenuated total reflectance Fourier transform infrared

### Discriminant analysis

**Figure-3:**
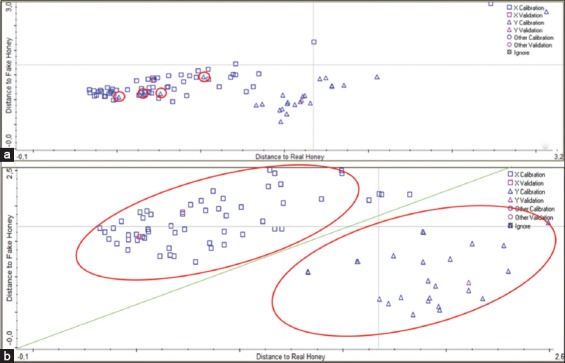
Discriminant analysis scatter plots for all of the samples of honey’s authenticity identification (a) 550-4000 cm^−1^, (b) 1600-1700 cm^−1^, 1175-1540 cm^−1^, 940-1175 cm^−1^, and 700-940 cm^−1^.

The different spectral region was applied to discriminant analysis, as shown in Figure-3. For identification purpose, the samples are divided into two groups: Real honey and fake honey. The spectra of real honey and fake honey are indicated by the square and triangle symbols, respectively. The calculated result of selected regions for identification purposes (real vs. fake honey samples) is shown in Table-2.

**Table 2:** Calculated result of selected region for identification purpose between real and fake honey.

Region	Performance index	Misclassified	Total samples
All (650-4000 cm^−1^)	88.7	7	85
1-5	91.8	-	85
2-5	91.8	-	85
Only 1 (2800-3000 cm^−1^)	90.0	3	85
Only 2 (1600-1700 cm^−1^)	90.1	6	85
Only 3 (1175-1540 cm^−1^)	90.4	6	85
Only 4 (940-1175 cm^−1^)	90.5	1	85
Only 5 (700-940 cm^−1^)	92	2	85
550-700 cm^−1^	87.7	7	85

As shown in Figure-3a, the spectrum of fake honey fell within the spectrum of real honey because the spectral region covered the whole area (the 500-4000 cm^−1^ region). The performance index for Figure-3a was 88.7, and there were seven samples that were misclassified. In Figure-3b, there are two regions separating real honey (square symbols) and fake honey (triangle symbols). The results are shown in Figure-3b – in which the performance index is 91.8 and no samples are misclassified – represent the best results following many trials. The spectral differences correspond to four regions: 1600-1700 cm^−1^, 1175-1540 cm^−1^, 940-1175 cm^−1^, and 700-940 cm^−1^. As shown in Table-2, the best performance index is obtained when we use regions 2–5.

**Figure-4:**
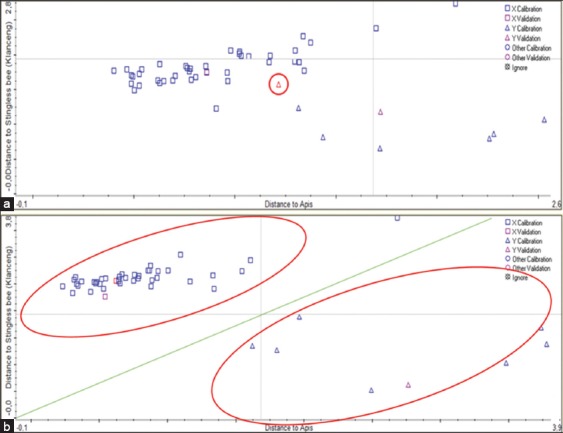
Discriminant analysis scatter plots for all of the samples of honey’s classification (a) 550-4000 cm^−1^, (b) 1600-1700 cm^−1^.

For classification purposes, the samples were divided into two classes: *Apis* spp. and *Tetragonula* spp. The spectra of honey from *Apis* spp. and *Tetragonula* spp. are indicated by the square and triangle symbols, respectively. Figure-4a and b are scatter plots of the discriminant analysis when the performance index is 88.2 and 95.4, respectively. In Figure-4a, we can see that a spectrum was misclassified (red circle), whereas no spectra were misclassified in Figure-4b. The results are shown in Figure-4b represent the best results following many trials. The calculated result of selected regions for classification purposes (*Apis* spp. vs. *Tetragonula* spp.) is shown in Table-3. The best performance index for the classification of honey is 95.4 and no samples were misclassified when we use region 2 only.

**Table 3:** Calculated result of selected region for classification purpose between *Apis* spp. and stingless bees.

Region	Performance index	Misclassified	Total samples
All (650-4000 cm^−1^)	88.2	1	50
1-5	93	-	50
2-5	93.1	-	50
Only 1 (2800-3000 cm^−1^)	92.9	1	50
Only 2 (1600-1700 cm^−1^)	95.4	-	50
Only 3 (1175-1540 cm^−1^)	93.2	-	50
Only 4 (940-1175 cm^−1^)	92	1	50
Only 5 (700-940 cm^−1^)	93.8	-	50
550-700 cm^−1^	92.4	1	50

The purpose of the trial is to precisely find where the spectral region differs. There are significant spectral differences between honey which are produced by *Apis* spp. and *Tetragonula* spp.

## Discussion

Ethanol in small quantities is a natural component of pure honey, and it is equivalent to <1% of the sugar content of honey [16]. Ethanol is produced by bacterial metabolism in the honey stomach of honey bees. Freshly harvested honey has a higher ethanol concentration due to the fermentation process in the honey bee stomach, and this concentration decreases overtime with evaporation. Previous study established that honey is considered a fermented food product due to the lactic acid bacteria (LAB) involved in the production process; a novel bacteria flora composed of LAB of the genera *Lactobacillus* and *Bifidobacterium*, which originated in the honey stomach of honey bees, was discovered [17]. The honey stomach represents an optimal niche for LAB because it is filled with nectar sugar and nutrients, and it operates at a fairly optimal temperature of 35°C in the hive [17,18].

Because the concentration of ethanol in honey depends on when the honey was harvested, some fake honey samples can contain ethanol while others contain no ethanol. Therefore, ethanol GC cannot be used as a valid method to test the authenticity of honey.

Testing of CO_2_ content revealed that honey from *Apis* spp. does not contain CO_2_, while honey from *Tetragonula* spp. does contain CO_2_, as characterized by a peak at a retention time of 2 min. This testing indicates that the honey samples produced by *Apis* spp. are original (naturally produced) because they do not contain CO_2_.

Honey produced by *Tetragonula* spp. contains a small amount of CO_2_ (0.100%). It is possible that *Tetragonula* spp. have a metabolic pathway that produces CO_2_, so the characteristics of the honey produced by these bees need to be further investigated. Because research on honey produced by *Tetragonula* spp. is very limited, it is difficult to control the quality of this honey as compared with the honey of *A. mellifera*, a more widely studied bee species [19].

The presence of ethanol and CO_2_ using GC methods cannot be used to identify the authenticity of honey. The results of this study showed the potential power of ATR-FTIR spectroscopy as an automated and highly sensitive method to differentiate between real and fake honey samples and between *Apis* spp. and *Tetragonula* spp. [20]. In this study, ATR-FTIR spectroscopy was used to compare honey samples based on their spectral differences in the 550-4000 cm^−1^ region. The crystal used in the ATR cells is made from materials that have a low solubility in water and a very high refractive index [21].

The discriminant analysis method successfully classified the honey samples based on their ATR-FTIR spectra [22]. There are four wavelength ranges that can best distinguish between the real and fake honey samples: 1600-1700 cm^−1^, 1175-1540 cm^−1^, 940-1175 cm^−1^, and 700-940 cm^−1^. For classification of the honey samples, the wavelength range of 1600-1700 cm^−1^ can best differentiate between *Apis* spp. and *Tetragonula* spp. Our proposed method is straightforward and suitable for the large-scale industrial monitoring of honey samples.

The discriminant analysis result shown the spectral differences correspond to four regions: 1600-1700 cm^−1^, 1175-1540 cm^−1^, 940-1175 cm^-1^, and 700-940 cm^−1^. The best performance index is obtained when we use regions 2–5. While the trials for regions 1–5 have the same scores as the performance index, region 1 can be ignored because it is a region of group frequency. The group frequency region of 2800-3000 cm^−1^ shows the absorption of C-C and C-H compounds; thus, this region can be ignored because it is common among all of the organic compounds.

The region of 800-1500 cm^−1^ corresponds to the absorption zones of the three major sugar constituents of honey: Fructose, glucose, and sucrose. The 750-900 cm^−1^ region is the anomeric region and is characteristic of the saccharide configurations. The bands in the 904-1153 cm^−1^ region are assigned to C-O and C-C stretching modes, and those around 1199-1474 cm^−1^ are due to the bending modes of O-C-H, C-C-H, and C-O-H angles. Negative bands were observed within the 1618-3635 cm^−1^ region. These bands are due to a lower water concentration in the honey sample compared with the reference employed and the fact that water presents an O-H stretching overtone at the corresponding wavelengths [23].

For classification purposes in the result shown, the best performance index for the classification of honey is 95.4, and no samples were misclassified when we use region 2 only. The bands in the region of 1600-1700 cm^−1^ had been previously assigned as amide I protein vibrations. Proteins are minor component in honey; however, they are used in detecting adulteration [24]. The previous studies revealed that pollen proteins could be used as a marker for taxonomic classification of honey. The bands appearing in the region of 1600-1700 cm^−1^ originated as a result of carbonyl group (C=O) and C≡C stretching, and this region was found to be related to phenolic molecules [25]. Phenolic compounds are linked to the biological origins of the nectar and pollen, and the species of the honey-producing bees [26-28]. However, water molecules show strong absorption between 1640 and 1650 cm^−1^, so the discrimination in this region can be explained by the difference in protein and moisture content, and water-carbohydrate interactions between sample groups [15].

Our several studies have been associated with bioactive compounds from stingless bee and *Apis* spp. honey with many other medicinal effects, as it was shown to have anti-inflammatory, antioxidant, antibacterial, and antidiabetic activity [29-31].

## Conclusion

We suggest a method to rapidly and accurately classify and authenticate honey using ATR-FTIR spectroscopy and chemometric method. This method successfully demonstrated to differentiate the authenticity and classification of honey based on the honey’s spectrum.

## Authors’ Contributions

SK, MG, MY, HH, and MS: Arranged, designed, and supervised the study. SK: Carried out sampling and laboratory analysis and wrote the first draft of the manuscript. SK, HH and YJY: Analyzed the data. MS, DKP, and MG: Contributed to the writing of the manuscript. MY, YJY, DKP, HH and MS: Jointly developed the structure and arguments for the paper. DKP, YJY, MY, and MG: Made critical revisions. All authors read and approved the final manuscript.
